# Bacterial Minicell‐Based Biohybrid Sub‐micron Swimmers for Targeted Cargo Delivery

**DOI:** 10.1002/advs.202505538

**Published:** 2025-06-27

**Authors:** Saadet Fatma Baltaci, Mukrime Birgul Akolpoglu, Irina Kalita, Victor Sourjik, Metin Sitti

**Affiliations:** ^1^ Physical Intelligence Department Max Planck Institute for Intelligent Systems 70569 Stuttgart Germany; ^2^ Stuttgart Center for Simulation Science (SC SimTech) University of Stuttgart 70569 Stuttgart Germany; ^3^ Max Planck Institute for Terrestrial Microbiology and Center for Synthetic Microbiology (SYNMIKRO) School of Medicine and College of Engineering 35043 Marburg Germany; ^4^ School of Medicine and College of Engineering Koç University Istanbul 34450 Turkey

**Keywords:** bacterial biohybrids, bacterial membrane vesicles, bacterial minicells, biohybrid microrobots, targeted drug delivery

## Abstract

Bacterial biohybrid microrobots possess significant potential for targeted cargo delivery and minimally invasive therapy. However, many challenges, such as biocompatibility, stability, and effective cargo loading, remain. Bacterial membrane vesicles, also referred to as minicells, offer a promising alternative for creating sub‐micron scale biohybrid swimmers (minicell biohybrids) due to their active metabolism, non‐dividing nature, robust structure, and high cargo‐carrying capacity. Here, a biohybrid system is reported that utilizes motile minicells, ≈400 nm in diameter, generated by aberrant cell division of engineered *Escherichia coli* (*E. coli*), for the first time. Achieving over 99% purification from their parental bacterial cells, minicells are functionalized with magnetic nanoparticles (MNPs) to enable external magnetic control. Minicell biohybrids are capable of swimming at an average speed of up to 13.3 µm s^−1^ and being steered under a uniform magnetic field of 26 mT. Furthermore, they exhibit a significantly high drug loading capacity (2.8 µg mL^−1^) while maintaining their motility and show pH‐sensitive release of anticancer drug doxorubicin hydrochloride (DOX) under acidic conditions. Additionally, drug‐loaded minicell biohybrids notably reduce the viability of SK‐BR‐3 breast cancer cells in vitro. This study introduces minicell biohybrids and establishes their potential as magnetically guided, drug‐loaded biohybrid systems for targeted therapies in future medical applications.

## Graphical abstract

Bacterial minicells, vesicles derived from engineered *E. coli*, offer promising sub‐micron scale biohybrid swimmers for targeted cargo delivery. Purified with over 99% efficiency while retaining motility, they are functionalized with MNPs for controlled movement. With high drug‐loading capacity and pH‐sensitive drug release, minicell biohybrids significantly reduce cancer cell viability, highlighting their potential for magnetically guided, minimally invasive therapies.

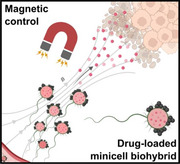

## 1 Introduction

Biohybrid microrobots (biohybrids), which integrate living microorganisms with artificial cargos or other body micro/nanomaterials, have been developed for over a decade to create miniaturized robotic platforms for various biomedical applications, including targeted cargo (e.g., drug) delivery, targeted therapy, minimally invasive surgery, diagnostics, and tissue engineering.^[^ ^1^, ^2^, ^3^ ^]^ Motile bacteria comprise the majority of microorganism‐based biohybrids due to their robust propulsion,^[^ ^4^ ^]^ ability to sense and respond autonomously to environmental changes,^[^ ^5^, ^6^ ^]^ and surface chemistries conducive to biophysical and chemical interactions for functional modifications.^[^ ^7^ ^]^ Despite their promise, potential clinical implementations of bacteria‐based biohybrids face challenges in terms of biocompatibility, biodegradability, structural stability, preserved motility, limited cargo‐carrying capacity, and navigating through physiological barriers or diseased tissues due to their size constraints.^[^ ^8^, ^9^ ^]^ To tackle these challenges, it is essential to develop new biohybrid designs that enhance the structural integrity, functionality, and biosafety while also leveraging the inherent properties of bacteria and the competence of synthetic body micro/nanomaterials. Currently, bacterial membrane vesicles, called minicells, could be an alternative for biohybrids, potentially overcoming most of the current limitations toward their potential clinical applications.

Minicells are spherical nanoscale bacterial membrane vesicles, typically ≈400 nm in diameter with 1–3 flagella,^[^ ^10^ ^]^ formed through aberrant cell division at polar sites of the parental cells.^[^ ^11^ ^]^ Although they are chromosome‐free, thus non‐living and non‐dividing entities, they maintain metabolic activities, such as energy production and protein synthesis.^[^ ^12^, ^13^ ^]^ Their smaller size may allow them to penetrate disease tissues more effectively than bacterial cells or biohybrids composed of larger microorganisms.^[^ ^2^, ^14^, ^15^ ^]^ Furthermore, minicells can transport a variety of therapeutic cargos and offer a high capacity for drug encapsulation and reduced drug leakage owing to the robust protection of bacterial cell membranes.^[^ ^16^ ^]^ This provides a significant advantage over conventional nanocarriers, such as liposomes, polymeric nanoparticles, and micelles.^[^ ^17^, ^18^, ^19^ ^]^ The unique nature of minicells not only allows for sustained self‐propulsion but also maintains the stability, biosafety, and functionality of the biohybrid structure harnessing these vesicles.

To date, numerous studies have demonstrated the potential of minicells derived from various bacterial strains for biomedical applications, such as immune system activation,^[^ ^20^ ^]^ cancer diagnosis,^[^ ^21^ ^]^ and especially the delivery of therapeutic agents. Delivered cargos include chemotherapeutic drugs,^[^ ^22^ ^]^ antibiotics,^[^ ^23^ ^]^ antigens,^[^ ^24^ ^]^ and synthetic si/shRNA.^[^ ^25^ ^]^ The administration of minicells, whether through direct application to the target region or via intravenous injection with cell‐specific targeting, has shown significant progress. However, challenges, particularly with cell‐specific targeting, remain in achieving precise and accurate targeting, effective localization, and accumulation within the target area.^[^ ^26^, ^27^ ^]^ Notably, no study has reported on the use of external control mechanisms, such as magnetic navigation, to guide minicells for active targeted drug delivery and improve drug accumulation. Furthermore, the minicells previously utilized for therapeutic delivery have generally lacked motility, which may stem from the use of non‐flagellated bacterial strains or flagellar damage caused by mechanical and chemical stress during the isolation process. These limitations have hindered the optimal delivery of therapeutic agents and reduced the overall effectiveness of minicell‐based treatments.

**Figure 1:**
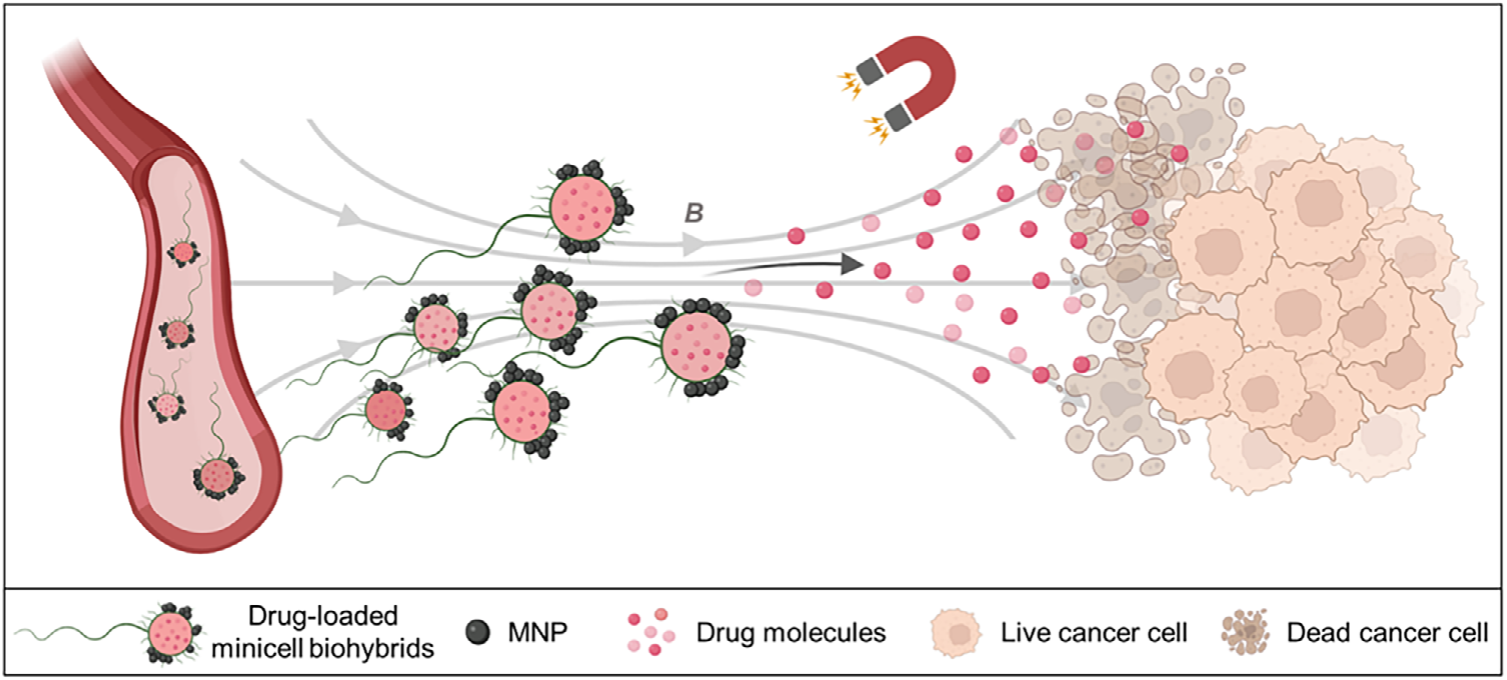
Conceptual illustration of sub‐micron scale minicell biohybrids for targeted therapy applications. The schematic depicts the minicell_DOX_ biohybrids conjugated with MNPs, which enable their magnetic steering to target cancer cells, where they deliver their drug payload for therapeutic activity.

Here, we developed a strategy employing genetically modified motile bacterial minicells, functionalized with drug molecules and MNPs to externally navigate them for targeted cancer therapy through a biohybrid approach (**Figure** **1**). First, we optimized the isolation process to achieve a high yield of flagellated minicells retaining their motility using both physical and biochemical methods. Then, we achieved the biohybridization of these minicells by conjugating them with streptavidin‐coated MNPs, enabling precise magnetic steering for targeted drug delivery. Furthermore, we characterized the cancer drug (DOX, as the model drug) carrying capacity of minicells and achieved precise magnetic control of drug‐loaded minicell (minicell_DOX_) biohybrids. Finally, we demonstrated their in vitro anticancer activity, by applying a pH‐triggered drug release mechanism that facilitates the selective release of the therapeutic agent in acidic tumor environments. This study highlights the potential of magnetically steerable minicell biohybrids for future therapeutic applications, which can remediate the limitations faced by the current bacterial biohybrid systems and minicell‐based therapies.

## 2 Results and Discussion

### 2.1 Production and Purification of Minicells

**Figure 2:**
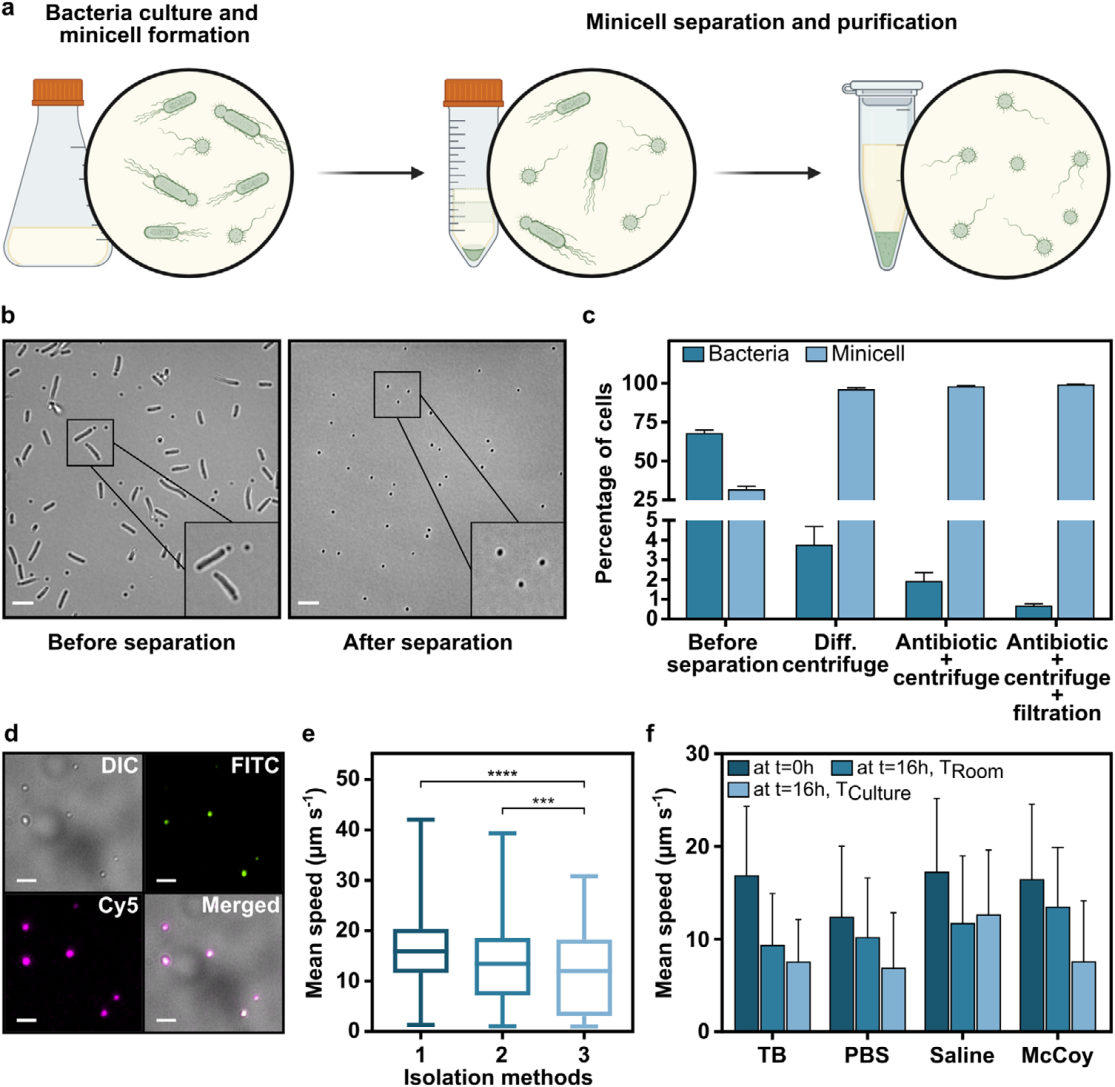
Production, purification, and motility characterization of minicells. a) A schematic of the minicell production during bacteria culture and isolation process. A more detailed protocol is illustrated in Figure S2 (Supporting Information). b) Optical microscopy images showing the bacterial culture and minicell suspension before and after separation. Scale bar: 5 µm. c) Percentage of parental bacteria and minicells, counted with widefield microscopy before and after the stepwise isolation process. d) Fluorescence microscopy images of minicells following purification. Green and magenta colors represented the GFP expression and biotin staining, respectively. Scale bar: 5 µm. DIC: Differential interference contrast, FITC: Fluorescein isothiocyanate, Cy5: Cyanine5. e) Mean swimming speed of minicells after different isolation methods: 1) differential centrifugation, 2) antibiotic treatment and centrifugation, and 3) antibiotic treatment and centrifugation followed by filtration. The box plots show the median (horizontal line) and upper and lower quartiles (box), along with the minimum and maximum (whiskers) speed values. For n = 5; ^***^ *P* < 0.0003, ^****^ *P* < 0.0001 ANOVA. f) Comparative motility analysis of minicells in different media over time stored at room and culture temperature (37 °C).

Minicells were generated by the genetically engineered strain of *E. coli* MG1655 with the deletion of the *min*CDE operon, which is responsible for mid‐cell positioning of the cell division septum.^[^ ^19^ ^]^ The minicell‐producing bacterial strain was evolved under selection for enhanced motility behavior on tryptone broth (TB) soft agar (Figure S1, Supporting Information) and further modified to express biotin‐binding peptides on its surface and cytoplasmic green fluorescent protein (GFP) for subsequent biohybridization of minicells and their fluorescence imaging, respectively. Minicells were produced during the bacterial growth until the optical density at 600 nm (OD_600_) reached ≈0.4, after which they were isolated from the parental cells (**Figure** **2**a; Figure S2, Supporting Information). Initially, we employed only differential centrifugation to separate minicells and achieved more than 96% reduction in the content of parental bacteria, representing a higher yield compared to previous studies.^[^ ^28^, ^29^ ^]^ Optical microscopy images were used to visualize both the bacterial culture and the minicell suspension before and after separation (Figure 2b).

To further purify the minicells, we treated the bacteria culture with ceftriaxone, an antibiotic that inhibits cell wall formation and therefore selectively targets dividing parental bacterial cells.^[^ ^30^ ^]^ This was followed by i) centrifugation and ii) an additional filtration step, resulting in improved isolation yields of 98.1% and 99.4% isolation, respectively (Figure 2c). The efficiency of minicell separation and purification was validated through a plate counting assay, which demonstrated continuous reduction of viable bacteria after each major isolation step (Figure S3, Supporting Information). The complete elimination of parental cells was achieved by extending the antibiotic treatment period up to 1 h. This adjustment significantly enhanced the purification process, ensuring the thorough removal of any residual parental cells while preserving the integrity of the isolated minicells.

Following the isolation process, we evaluated whether the minicells retained key functional properties from the parental cells: their ability to express proteins (biotin‐binding peptides and GFP) with stable expression levels and maintain motility. Since antibiotic treatment could potentially affect protein synthesis,^[^ ^31^ ^]^ it was essential to confirm both the presence of biotin‐binding peptides on the minicell surface and the preserved expression of GFP to ensure their compatibility with biohybridization. To assess the effect of antibiotic treatment on protein expression in minicells, we measured the fluorescence intensity of biotin staining detected by fluorescently tagged (Cy5) streptavidin and GFP of both antibiotic‐treated and untreated minicells. No significant changes in fluorescence intensity were observed for either biotin staining (Figure S4a, Supporting Information) or GFP expression following antibiotic treatment (Figure S4b, Supporting Information), indicating that protein expression levels remained consistent. Overall, minicells isolated through both differential centrifugation and antibiotic treatment exhibited clear fluorescence signals corresponding to GFP expression and biotin staining (Figure 2d). These findings demonstrate that the antibiotic‐based purification process did not compromise the protein expression in minicells, thereby preserving their functionality for subsequent biohybridization.

Furthermore, we investigated the functional stability of minicells under different environmental conditions, focusing on their metabolic activity in response to decreasing pH levels, which is critical for pH‐sensitive drug release from minicell biohybrids. Since GFP expression serves as a key metabolic marker for imaging and localization in such biomedical applications, we examined whether an acidic environment affects GFP expression levels. For that, we measured the fluorescence intensity of GFP‐expressing minicells incubated overnight in culture media at different pH levels: acidic (pH 4.6) and slightly acidic (pH 6.5) and compared it to those incubated in culture medium at neutral pH. Our results showed no significant changes in fluorescence intensity across different pH conditions (Figure S5, Supporting Information), suggesting that the acidic environment did not noticeably affect protein expression in minicells. The findings highlight the robustness of minicells in biohybrid systems designed for controlled release in acidic conditions.

### 2.2 Characterization of Minicell Motility

A recent study,^[^ ^10^ ^]^ which introduced motile and chemotactic minicells, paved the way for advanced strategies that combine motile minicells with existing therapeutic delivery systems and biohybrid microrobot designs. The ability of minicells to move actively and navigate toward specific targets, such as tumor sites, enhances their effectiveness as drug carriers and broadens their potential for biomedical applications. Given the importance of this motile behavior for the overall efficacy of biohybrid systems, we conducted a detailed characterization of the motility of minicells isolated using different methods. The average swimming speed of minicells separated by only differential centrifugation was measured at 16.0 ± 6.9 µm s^−1^, corresponding to ≈40 body lengths per second (BLPS). When the minicells were isolated with antibiotic treatment followed by i) centrifugation and ii) an additional filtration, their average swimming speeds decreased to 13.4 ± 7.6 µm s^−1^ and 11.6 ± 7.8 µm s^−1^, respectively (Figure 2e; Movie S1, Supporting Information). Despite the reduction in their motility, the minicells still maintained fast swimming speeds relative to their body size, with the slowest measured at ≈30 BLPS. Additionally, minicells demonstrated robust swimming ability, exhibiting resilience to high centrifugation forces and multiple repeated cycles without significant changes in their average speed (Figure S6, Supporting Information). This sets them apart from other free‐swimming organisms, making them a compelling candidate as the cellular propeller of biohybrids.^[^ ^1^, ^32^ ^]^

We further assessed the motility of minicells in various liquid media, including physiological solutions, such as phosphate‐buffered saline (PBS), saline, and the cell culture medium McCoy's 5A. Also, we compared their motility to that observed in the bacterial growth medium (TB) (Figure 2f). Initially, no significant decrease in motility was observed in saline or cell culture medium, with average swimming speeds of 17.3 ± 7.9 µm s^−1^ and 16.7 ± 7.5 µm s^−1^, respectively, compared to 17.4 ± 6.6 µm s^−1^ in bacterial growth medium. However, a notable effect on motility was observed in PBS, with the average swimming speed at 12.4 ± 7.6 µm s^−1^. Then, we analyzed the motility of minicells over time at room temperature and culture temperature (37 °C). After overnight incubation, the motility of minicells decreased at both temperatures. A smaller decrease in average speeds was observed at the lower temperature, which might slow down metabolic processes, thus preserving energy and allowing the minicells to maintain a certain level of motility over a longer period. For instance, the average swimming speeds of minicells in TB were recorded as 9.4 ± 5.5 µm s^−1^ and 7.6 ± 4.5 µm s^−1^ at the room and culture temperature, respectively. A similar trend in motility was noted across all media, with average swimming speeds dropping to nearly half of their initial values except for saline. In saline, the minicells maintained a significantly higher average swimming speed of 12.7 ± 6.9 µm s^−1^ and 11.8 ± 7.2 µm s^−1^, which were at least 35% greater than those recorded in the other media. The relatively high motility in saline is particularly important since it is commonly used in the medical administration of drugs or drug carriers.^[^ ^33^ ^]^

Lastly, we conducted additional experiments to analyze the effect of the decrease in pH on minicell motility over time. Even though we observed a slight decrease in the average swimming speed of minicells in slightly acidic and acidic conditions (pH 6.5 and pH 4.6, respectively) compared to neutral pH, the effect of acidity on minicell motility, both at the start of the experiment and after overnight incubation, was not statistically significant (Figure S7, Supporting Information). Notably, minicells maintained a level of motility comparable to normal bacterial cells under acidic conditions,^[^ ^34^ ^]^ highlighting their resistance to environmental stress relative to bacteria. These results demonstrated that minicells can retain motility in both physiologically relevant and highly acidic conditions, further supporting their potential for biomedical applications.

### 2.3 Production of Minicell Biohybrids and their Magnetic Steering

**Figure 3:**
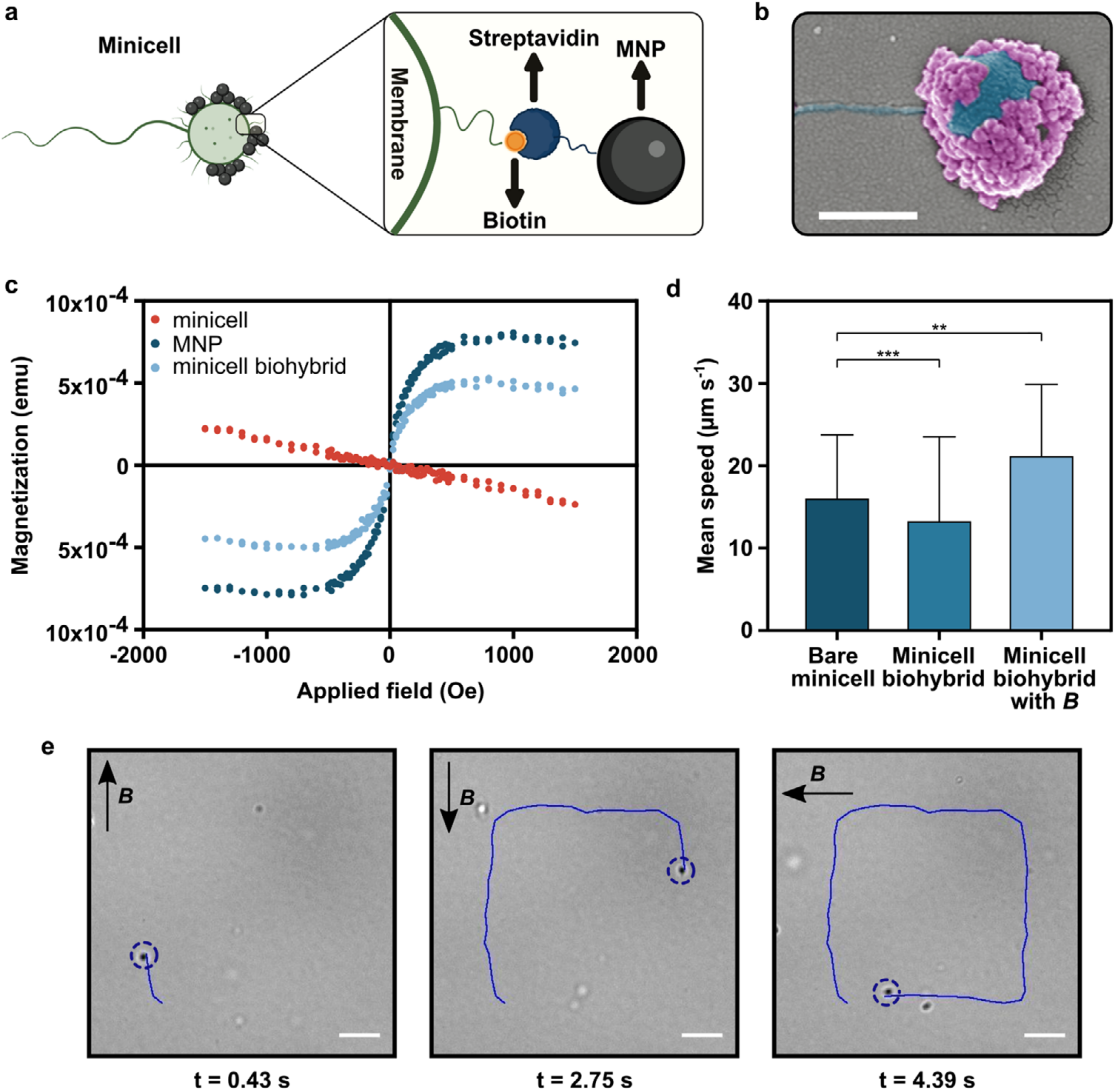
Characterization of minicell biohybrids and their magnetic steering. a) A schematic illustration of minicell biohybrids conjugated with magnetic nanoparticles via biotin‐streptavidin interaction. b) A representative scanning electron microscopy (SEM) image of a minicell biohybrid carrying magnetic nanoparticles. The image was pseudo‐colored for visualization purposes; pink indicates MNPs, and blue indicates the minicell with flagellum. Scale bar: 400 nm. c) Magnetic behavior of minicells before and after biohybridization. The figure shows the magnetic hysteresis loops obtained at room temperature using vibrating sample magnetometry (VSM) for bare minicells, MNPs, and minicell biohybrids. d) 2D mean swimming speeds of bare minicells and minicell biohybrids with and without the applied uniform magnetic field of 26 mT. The error bars represent the SD of the mean for n = 5; ^**^ *P* = 0.0067, ^***^ *P* = 0.0003 ANOVA. e) Magnetic steering of minicell biohybrids by electromagnetic coil setup under a uniform magnetic field changing direction by 90°. Scale bar: 5 µm.

The fabrication of biohybrid micro/nanorobots has been performed through a variety of integration strategies, ranging from chemical and physical interactions to cellular internalization.^[^ ^35^, ^36^, ^37^ ^]^ Among these, we successfully employed the biotin‐streptavidin interaction between the biotinylated minicells and streptavidin‐functionalized MNPs to create minicell biohybrids (illustrated in **Figure** **3**a), a gentle yet robust method that preserves the motility and the taxis ability of the organism. This approach is particularly advantageous when bacteria are engineered to display biotin on their cell surface, enabling high‐efficiency fabrication without the need for lengthy multistep processes that could potentially damage the fragile and limited number of minicell flagella.^[^ ^10^, ^38^ ^]^

The successful magnetization of motile minicells was confirmed through SEM imaging of minicell biohybrids (Figure 3b). Additionally, VSM measurements were performed to characterize the magnetization properties of minicell biohybrids. Minicells, which naturally exhibit diamagnetic properties inherited from bacteria,^[^ ^39^ ^]^ acquired the magnetic properties of superparamagnetic nanoparticles after conjugation, with a saturation magnetization of 519.16 × 10⁻⁴ emu for minicell biohybrids, compared to 797.88 × 10⁻⁴ emu for MNPs alone (Figure 3c). Lastly, we analyzed the zeta potential of minicell before and after functionalization for further characterization of minicell biohybrids. Minicells naturally exhibit a negative electrostatic surface charge, which we measured as ‐17.7 ± 1.1 mV at neutral pH (Table S1, Supporting Information). After conjugation with MNPs, the net charge of minicells slightly decreased, and the zeta potential of minicell biohybrids was measured as ‐20.8 ± 0.5 mV. While this suggests that functionalization with MNP conjugation may have influenced the surface charge, it did not significantly change the overall electrostatic properties of minicells. These results, along with the SEM images, fully confirm the successful attachment of the MNPs to minicells, leading to a measurable magnetic response under an applied field without a notable impact on their electrostatic properties. We subsequently analyzed the motility of minicell biohybrids and compared it to that of bare minicells to evaluate the impact of particle attachment on their swimming behavior. The average swimming speed of free‐swimming minicell biohybrids was measured at 13.3 ± 10.0 µm s^−1^, whereas under a uniform magnetic field of 26 mT, generated by two circular permanent magnets along one direction, the minicell biohybrids exhibited an increased average speed of 21.2 ± 8.6 µm s^−1^ (Figure 3c; Movie S2, Supporting Information). Despite the change in the swimming speed and distribution of minicells after biohybridization, the overall motion pattern of the minicells was not affected by particle conjugation, as demonstrated by their 2D swimming trajectories (Figure S8, Supporting Information). The magnetic alignment significantly enhanced the motility of minicell biohybrids by facilitating their swimming in a given direction continuously with reduced cell rotation and minimized angular changes during the run‐and‐tumble motion, similar to other magnetic biohybrid systems.^[^ ^40^, ^41^ ^]^ We further demonstrated the magnetic steering ability of minicell biohybrids by applying a 2D uniform magnetic field generated by an electromagnetic coil setup. The minicell biohybrids responded precisely to alterations in the magnetic field direction, resulting in a well‐defined square‐shaped swimming trajectory (Figure 3d; Movie S3, Supporting Information) with a normal distribution (Figure S8, Supporting Information). This study is the first to successfully demonstrate the magnetization and controlled steering of minicells. This achievement represents a key advancement, as it allows for precise directional control of minicell biohybrids, potentially improving targeted drug delivery by ensuring accurate localization and reducing nonspecific distribution.

### 2.4 Minicell Biohybrids as Active Drug Delivery Vehicles

Bacterial minicells, discovered nearly half a century ago, have been extensively studied over the past two decades for their potential as carriers of therapeutics, particularly in cancer therapy.^[^ ^19^ ^]^ While advancements have been made to increase their therapeutic competence by enhancing their specificity for cancer cells through ligand‐ or antibody‐mediated targeting, the efficacy of these approaches is often constrained by limitations in targeting accuracy and localization, leading to undesired off‐target effects.^[^ ^42^ ^]^ However, creating motile minicell biohybrids and utilizing them as active drug carriers with magnetic targeting could minimize these off‐target effects by accurately guiding minicell biohybrids to the target area.

**Figure 4:**
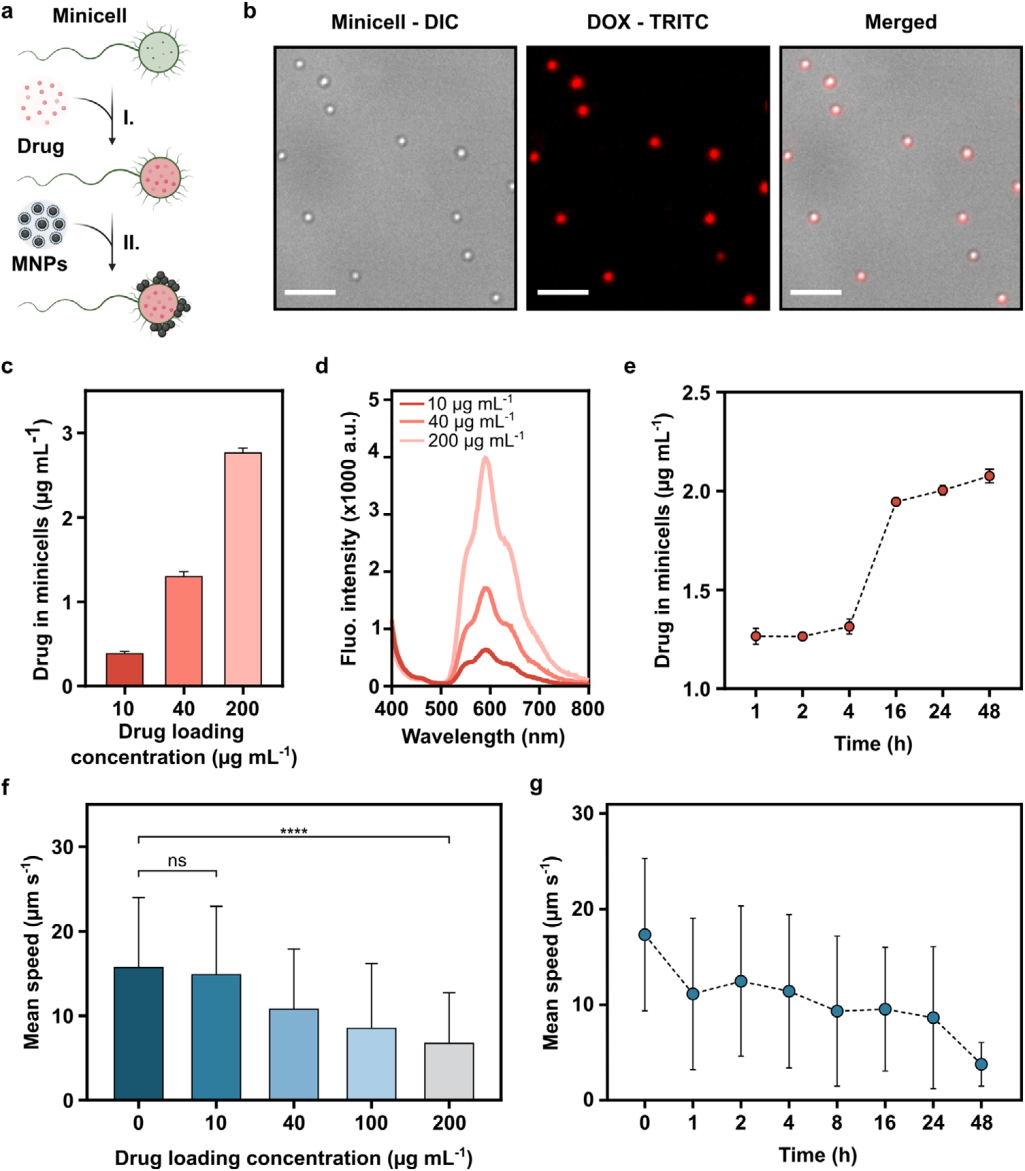
Characterization of drug‐carrying capacity and motility of minicell_DOX_ biohybrids. a) A schematic of the preparation of minicell_DOX_ biohybrids. I. Drug loading into minicells, II. Conjugation of MNP onto minicell_DOX_. b) Fluorescence microscopy images of minicell_DOX_ incubated with 40 µg mL^−1^ DOX for 4 h. Scale bar: 5 µm. c) Quantification of DOX encapsulation within minicells after incubation with varying drug concentrations for 4 h. d) The fluorescence spectrum of DOX encapsulated within minicells at given concentrations after 4 h. e) Time‐dependent drug loading into minicells with 40 µg mL^−1^ DOX over 48 h. f) Motility analysis of minicell_DOX_ after incubation with various concentrations of DOX for 4 h. g) The effect of the drug loading period on the motility of minicells. DOX was loaded at the concentration of 40 µg mL^−1^. The error bars represent the SD of the mean for n = 3; ns: not significant, ^****^ *P* < 0.0001 ANOVA.

Within this context, we developed magnetically controlled minicell_DOX_ biohybrids to improve the targeting precision of minicell‐based drug delivery, as illustrated in (**Figure** **4**a). To ensure optimal performance, we first investigated the drug‐carrying capacity of biotinylated minicells and assessed the effect of drug loading on their motility. Minicells uptake DOX by diffusion across the lipid bilayer, which is supported by the presence of non‐specific porin channels in their outer membranes that allow various solutes,^[^ ^43^ ^]^ including DOX, to penetrate and become encapsulated, instead of merely adhering to the minicell surface.^[^ ^22^, ^44^, ^45^ ^]^ Initially, minicells were incubated with low, medium, and high concentrations of the drug, corresponding to 10, 40, and 200 µg mL^−1^ DOX solution, for 4 h. After incubation with the drug, all of the minicells displayed the autofluorescence of DOX (excitation, 470 nm/emission, 585 nm), confirming DOX internalization within minicells rather than surface adsorption (Figure 4b). The amount of DOX encapsulated within the minicells was measured at 0.40 ± 0.01, 1.32 ± 0.04, and 2.78 ± 0.04 µg mL^−1^, respectively, with distinct peaks observed in the fluorescence spectrum, even at the lowest drug concentration (Figure 4c,d). Notably, the drug‐loading capacity of the biotinylated minicells exceeded that of previously studied surface‐modified *E. coli*‐derived minicells.^[^ ^14^ ^]^ Next, we analyzed the drug‐loading capacity of biotinylated minicells over different loading times by incubating them with 40 µg mL^−1^ DOX. In the first 4 h of incubation, the amount of drug encapsulated within the minicells barely increased from 1.27 ± 0.04 µg mL^−1^ to 1.32 ± 0.04 µg mL^−1^, while the amount of drug significantly increased to 1.95 ± 0.09 µg mL^−1^ after overnight incubation (Figure 4e). To determine whether drug loading continues beyond this point, we extended the incubation to 24 and 48 h. The amount of encapsulated DOX increased slightly to 2.00 ± 0.02 µg mL⁻¹ at 24 h and 2.07 ± 0.04 µg mL⁻¹ at 48 h, showing a decelerating trend and indicating possible onset of saturation after 48 h. Although the drug loading curve does not reach a clear plateau at 48 h, we did not extend the incubation further, as minicell motility greatly reduced to 3.8 µm s⁻¹ after 48 h of drug exposure.

Our findings suggest that minicell biohybrids demonstrate an enhanced drug‐carrying capacity when incubated with high concentrations of DOX over extended periods. However, the potential impact of the drug loading process on the motility of the minicell biohybrids must be carefully considered, as it may influence the optimal selection of loading time and concentration. No prior studies have systematically examined the motility of minicell_DOX_ or provided data on their swimming speeds, which is a critical factor for effective targeted drug delivery. To fulfill this gap, we conducted the first comprehensive motility characterization of minicell_DOX_ and minicell_DOX_ biohybrids, focusing specifically on how drug payload and incubation time influence their swimming behavior. Minicells, initially exhibiting an average swimming speed of 16.04 ± 7.45 µm s ^−1^, were incubated with varying concentrations of DOX (10, 40, 100, and 200 µg mL^−1^). Following a 4 h incubation period, the average swimming speeds of minicell_DOX_ were measured as 15.0 ± 7.9 µm s^−1^, 10.9 ± 6.9 µm s^−1^, 8.7 ± 7.5 µm s^−1^, and 6.9 ± 5.8 µm s^−1^, respectively. Whereas the average swimming speed of the control group remained almost constant at 15.9 ± 8.1 µm s^−1^ (Figure 4f). The observed decrease in swimming speed with higher concentrations of DOX indicates that the drug affects the motility of minicells, which could be due to the toxic effects of DOX at higher concentrations, potentially impairing the structural integrity or energy metabolism of the cells as shown in studies with bacteria.^[^ ^46^, ^47^ ^]^ Furthermore, we investigated the change in minicell motility over time by incubating biotinylated minicells with 40 µg mL^−1^ DOX. The results demonstrated a gradual decrease in the average swimming speed of minicells incubated with 40 µg mL^−1^ DOX over time recorded as 11.2 ± 7.9 µm s^−1^, 12.5 ± 7.9 µm s^−1^, 11.4 ± 8.0 µm s^−1^, 10.3 ± 8.1 µm s^−1^, 9.5 ± 6.5 µm s^−1^ and 8.64 ± 7.45 µm s^−1^ at 1, 2, 4,8, 16 and 24 h, respectively (Figure 4g; Movie S4, Supporting Information). This trend in the swimming speed of minicell_DOX_ closely mirrored the pattern observed in the control groups without DOX (Figure S9, Supporting Information). Then, a drastic decline in the motility was observed after 48 h of incubation, with the average swimming speed decreasing to 3.77 ± 2.29 µm s^−1^, which indicates a significant impact of prolonged DOX exposure on the swimming behavior of minicells (Figure 4g).

Considering the need for effective drug loading, motility retention, and simplicity of experimental procedures, we developed a method to fabricate minicell_DOX_ biohybrids. We used biotinylated minicells, incubated overnight with 40 µg mL⁻¹ DOX solution, to achieve optimal drug loading and preserve motility. Under free‐swimming conditions, the minicell_DOX_ biohybrids exhibited an average swimming speed of 5.7 ± 4.6 µm s^−1^ without the application of the magnetic field. When exposed to a uniform magnetic field of 26 mT, their speed increased to 12.4 ± 4.4 µm s^−1^ (Figure S10, Supporting Information), demonstrating controlled, unidirectional movement (Movie S5, Supporting Information). This indicates that the combination of drug encapsulation and particle conjugation did not impair the swimming capabilities of the minicell biohybrids under constant magnetic alignment. The ability of minicell_DOX_ biohybrids to navigate efficiently could improve the therapeutic outcomes by influencing their distribution within the body and potential localization on the site of action. It is worth highlighting that the motile nature of our minicell‐based biohybrid system provides a distinct advantage compared to passive nanosystems, such as liposomes and synthetic particles at similar length scales, in the context of drug‐delivery applications.^[^ ^48^ ^]^ The active propulsion of minicells, combined with magnetic control, could lead to more effective drug delivery, particularly in complex physiological environments and hard‐to‐reach tissues. Furthermore, compared to other biohybrid microrobots, such as those created from living and dividing bacteria or microalgae, our minicell biohybrids offer the advantage of being non‐proliferative, thus preventing drug dilution and minimizing the risk of uncontrolled growth and enhancing the safety profile of the system for future clinical applications.

### 2.5 In vitro Anticancer Activity Characterization of Minicell_DOX_ Biohybrids

**Figure 5:**
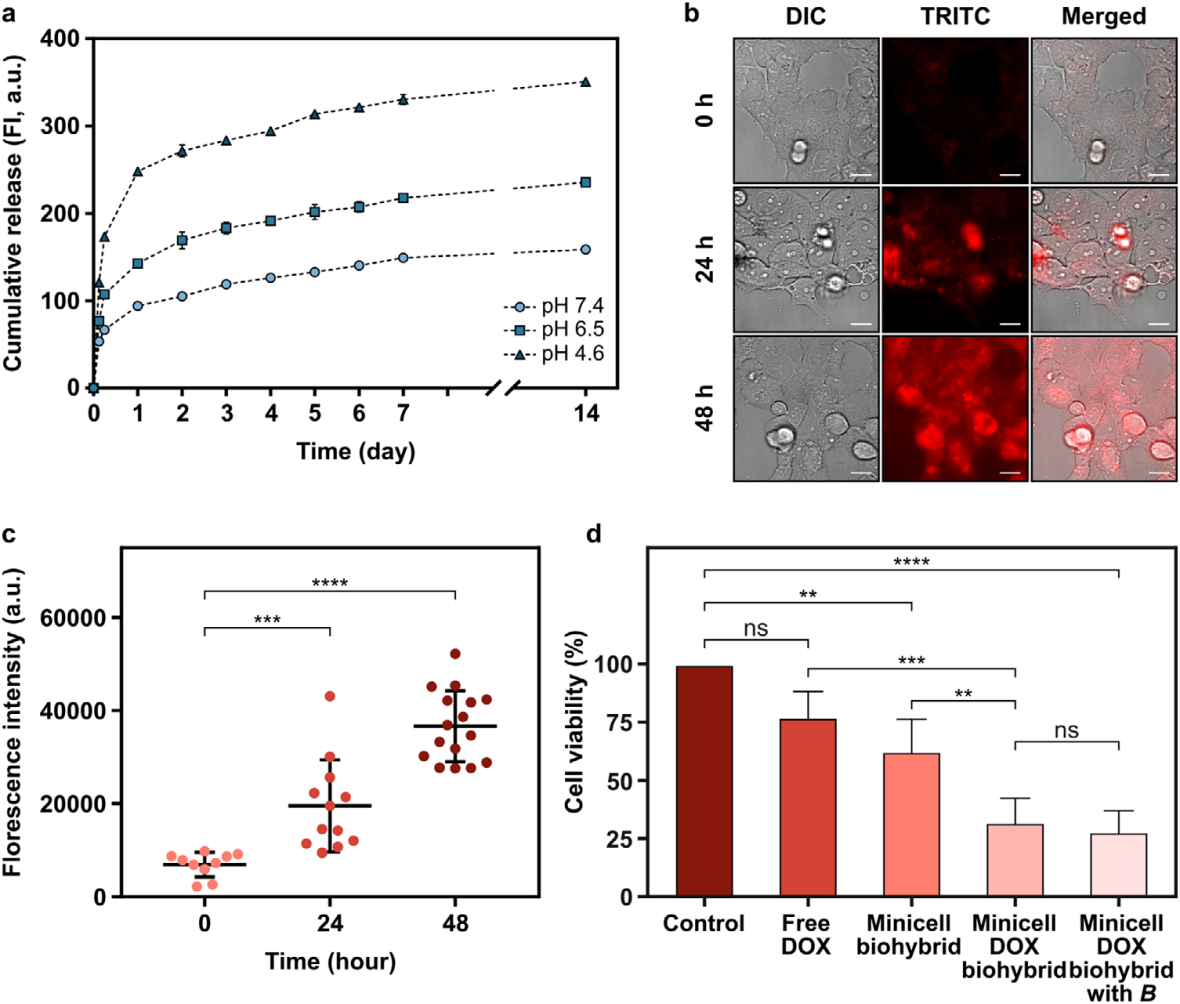
Drug release characterization and therapeutic activity of minicell_DOX_ biohybrids. a) pH‐responsive cumulative release of DOX encapsulated in minicells for a two‐week period at pH 4.6, 6.5 and 7.4. b) Fluorescence microscopy images of in vitro drug uptake of SK‐BR‐3 cells incubated with minicell_DOX_ for 0, 24 and 48 h. DOX inside the cells was shown by the red color. Scale bar: 20 µm. c) Quantification of mean DOX fluorescence intensity on SK‐BR‐3 cells over time. The mean ± SD for n ≥10, ^***^ *P* = 0.0010, ^****^ *P* < 0.0001, t‐test. d) The percent viability of SK‐BR‐3 cells incubated with free DOX, minicell biohybrids and minicell_DOX_ biohybrids with and without magnetic actuation at pH 6.5 for 24 h. The error bars represent the SD of the mean for n = 3; ns: not significant, ^**^ *P* < 0.0018, ^****^ *P* < 0.0001 ANOVA. In all experiments shown in this figure, drug loading into minicells was performed by overnight incubation of minicells with DOX at a concentration of 40 µg mL^−1^.

The minicell_DOX_ biohybrids, with their natural motility and magnetic controllability, present a promising approach for targeted drug delivery. They could provide improved precision in directing therapeutic agents, potentially increasing bioavailability, reducing required doses, and enhancing drug retention, making them an effective alternative to conventional drug delivery systems. The pH‐sensitive release of DOX from the engineered minicells is similar to mechanisms found in other drug delivery systems having lipid bilayers. Upon exposure to the acidic microenvironments common in tumor tissues, the minicell membrane integrity may be compromised, leading to the controlled release of DOX.^[^ ^14^ ^]^ To broadly evaluate the therapeutic potential of minicell biohybrids in vitro, we first examined the drug release profile from DOX‐loaded minicells over a two‐week period at different pH levels: 7.4, 6.5, and 4.6 (**Figure** **5**a). The results showed that at neutral pH (7.4), the cumulative release of DOX reached a maximum of only 5.4%, indicating that the minicells exhibit minimal drug leakage under physiological pH conditions. Our finding aligns with previous results showing unidirectional drug transfer in minicells derived from different bacterial strains in a distinct medium yet under the same pH condition as highlighted.^[^ ^49^ ^]^ Notably, drug release significantly accelerated as the acidity of the environment increased with the highest release observed at pH 4.6, where within the first 24 h, the amount of DOX released was nearly double that at pH 6.5 and triple that at pH 7.4. The release profile exhibited an initial rapid phase within the first 6 h (Figure S11, Supporting Information), followed by a sustained release over 48 h. The pH‐responsive drug release of minicells enhances the therapeutic efficiency of minicell biohybrids, ensuring that the drug is preferentially released in the acidic environments typically found in tumor tissues and retained at physiological conditions. Additionally, cancer cells can internalize bacterial minicells through receptor‐mediated endocytosis or phagocytosis^[^ ^22^, ^25^, ^50^ ^]^ and ligand‐driven non‐endocytic route.^[^ ^14^ ^]^ Once internalized, minicells are degraded in the intracellular environment, facilitating the complete release of the therapeutic cargo and thereby maximizing the therapeutic efficacy of minicell biohybrids. Localizing these biohybrids to the tumor site through magnetism‐assisted targeting could further improve treatment outcomes by concentrating the drug‐loaded biohybrids at the tumor site while potentially minimizing systemic toxicity, as demonstrated in a previous study using bacterial biohybrids decorated with drug‐loaded nanoliposomes.^[^ ^40^ ^]^

In parallel, we demonstrated the anticancer potential of minicell biohybrids by investigating drug uptake in SK‐BR‐3 cancer cells co‐cultured with minicell_DOX_ over a 48 h period. Confocal microscopy images illustrated the spatial distribution of minicell_DOX_ and the interaction between DOX and cancer cells (Figure S12, Supporting Information). In addition, the fluorescence microscopy images (Figure 5b) and quantitative intensity measurements (Figure 5c) revealed a time‐dependent increase in DOX fluorescence, which indicated a sustained drug release from minicells and subsequent internalization by SK‐BR‐3 cells over a 48 h period. Compared to free DOX (Figure S13, Supporting Information), which rapidly entered and accumulated in the cells, minicell_DOX_ exhibited a more gradual and sustained release profile. This controlled release is likely to enhance anticancer efficacy by enabling prolonged drug accumulation within tumor tissue and facilitating more efficient cellular uptake over time.^[^ ^51^, ^52^ ^]^ The therapeutic effect of this sustained delivery was further validated using Live/Dead staining, which assesses cell viability based on membrane integrity. Fluorescence images showed an increase in the number of dead cells (represented by red color) within 48 h (Figure S14, Supporting Information), indicating that drug‐loaded minicells induce cell death characterized by membrane disintegration over time.

Lastly, we assessed the viability of breast cancer cells treated with minicell biohybrids, minicell_DOX_ biohybrids (with and without magnetic field exposure), and free DOX at pH 6.5 using a metabolic activity‐based assay to provide a more comprehensive understanding of treatment outcomes (Figure 5d). After 24 h of incubation, the viability of cancer cells treated with free DOX was measured as 77%. However, when cancer cells were exposed to minicell_DOX_ biohybrids with and without magnetic control, the cellular viability dropped to 32% and 28%, respectively. Magnetic steering had only a slight additional decrease in cell viability, likely due to the use of a uniform magnetic field that provided directional guidance but did not generate sufficient rotational motion or mechanical stimulation to enhance cytotoxicity further.^[^ ^53^, ^54^ ^]^ Overall, minicell_DOX_ biohybrids exhibited a significantly greater impact on the metabolic activity of cancer cells compared to both free DOX and minicell biohybrids alone. The higher cytotoxicity of drug‐loaded minicell biohybrids compared to free DOX can be attributed to both the inherent anticancer activity of the bacterial origin of minicells^[^ ^11^, ^55^ ^]^ and the mechanism of action of DOX.^[^ ^56^, ^57^ ^]^ The combination of minicell biohybrids and the chemotherapeutic drug resulted in a powerful synergistic effect, which together improves the overall therapeutic outcome. These findings highlight the potential of minicell biohybrids in cancer treatment, as their biological origin enables multi‐modal therapeutic payload delivery, while their motility and magnetic controllability offer opportunities for enhanced localization. It is important to note that in this study, SK‐BR‐3 cells were chosen as a well‐established and widely used breast cancer model to demonstrate proof‐of‐concept for the drug delivery and cytotoxic efficacy of the minicell biohybrids. However, further studies in additional cancer cell lines are needed to confirm the broader applicability of this platform. Ongoing research and clinical trials will be essential to fully understand and optimize this combination for broader use in cancer therapy.

## 3 Conclusion

Here, we introduced a sub‐micron scale biohybrid swimmer design comprising motile and drug‐loaded bacterial minicells functionalized with magnetic nanoparticles. Apart from their smaller size which has the potential to overcome biological barriers more easily, the self‐propulsion of minicells promotes active motility, which is absent in synthetic drug‐loaded passive particles. While minicell biohybrids were precisely steered by the externally applied magnetic field, they retained their motility and structural integrity thanks to the non‐proliferation of minicells over an extended application period, unlike other magnetically controlled bacteria‐based biohybrids. The biohybrid structure presented here offers a unique approach by harnessing the benefits of motile minicells. Their versatile nature allows for functionalization and efficient drug encapsulation through a single‐step and non‐invasive biohybridization, eliminating the need for additional carriers to facilitate drug delivery. Furthermore, the minicell_DOX_ biohybrids demonstrated potent anticancer activity in vitro with minimal drug leakage and pH‐sensitive drug release that targets cancerous environments.

Although minicell biohybrids show significant potential for targeted drug delivery in cancer therapy, further investigations are required. Their interactions within the complex tumor microenvironments need to be thoroughly understood to enhance their performance, particularly considering the smaller size of minicell biohybrids, which could offer advantages in penetrating tumor tissues under magnetic guidance. Additionally, the collective motion of minicell biohybrids and their swarm dynamics, which could ease the navigation through complex biological environments or in vivo monitoring,^[^ ^58^ ^]^ has yet to be studied. Last but not least, it is essential to balance the biosafety and therapeutic efficacy of these biohybrids following a systemic administration, while thoroughly evaluating their immunostimulatory activities. Overall, this biohybrid system uses the efficient drug‐carrying capacity of bacterial minicells and magnetic control to deliver drugs precisely, setting the foundation for developing versatile and targeted future drug delivery systems.

## 4 Experimental Section

### Bacterial Strain and Plasmids

The derivative of *E. coli* MG1655 with the deletion of the entire *minCDE* operon was used in this study. The deletion of the operon was conducted using λ Red recombination followed by kanamycin cassette excision via FLP recombination. The motility of the strain was enhanced via experimental evolution performed over 14 days in 0.27% soft agar TB plates as described previously.^[^ ^59^ ^]^ The evolved strain was next transformed with the plasmid pOS233, encoding biotinylated autotransporter antigen 43 (Ag43), and pOS239, allowing for the arabinose‐inducible expression of GFP.^[^ ^38^ ^]^

### Bacterial Growth Conditions and Minicell Production

Bacterial cells stored at ‐80 °C in a 50% glycerol stock were first grown on the Luria–Bertani (LB) agar plates (15 g L^−1^ agar, 5 g L^−1^ NaCl, 40 g L^−1^ tryptone and 5 g L^−1^ yeast extract; LB Broth with agar‐Lennox, Sigma‐Aldrich) supplemented with 100 µg mL^−1^ ampicillin and 50 µg mL^−1^ kanamycin (Sigma‐Aldrich) as single colonies. Before each experiment, single colonies were cultivated overnight in 5 mL TB medium[(10 g L^−1^ tryptone (Santa Cruz Biotechnology Inc.) and 5 g L^−1^ NaCl (Sigma‐Aldrich) at pH 7.0] supplemented with 100 µg mL^−1^ ampicillin and 50 µg mL^−1^ kanamycin (Sigma–Aldrich) at 37 °C and 200 rpm. For minicell production, the overnight culture was transferred into 20 mL of TB medium containing the antibiotics and biotin (2 µm, Sigma–Aldrich) at a 1:100 ratio and incubated at 37 °C and 220 rpm for 30 min. The expression of biotinylated Ag43 and GFP was induced by adding isopropyl‐β‐d‐thiogalactopyranoside (250 µM, Sigma–Aldrich) and L (+)‐Arabinose (0.005% w/v, Sigma–Aldrich) into the culture, which was further cultivated for ≈5‐6 h until the optical density at 600 nm (OD_600_) reached 0.35–0.4, measured using a microplate reader (TECAN Infinite M Plex).

### Minicell Separation and Purification

Once the optical density of the culture reached 0.35–0.4, minicell isolation was carried out using two separate methods based on differential centrifugation and antibiotic treatment. The bacteria culture (20 mL) was first centrifuged at 3220 g, 4 °C for 15 min. For the differential centrifugation method, the upper 5 mL of the supernatant was discarded, and ≈6 mL of the middle fraction was collected. It was evenly distributed into Eppendorf tubes for the second centrifugation at 3220 g at 4 °C for 5 min. The upper 0.5 mL of the supernatant was removed, and the remaining 0.5 mL from the middle fraction was subjected to a high‐speed centrifugation step at 40 000 g at room temperature for 5 min. ≈450 µL of the supernatant was carefully removed, and the minicells were gently resuspended in the remaining volume of medium for subsequent steps. For the purification of minicells based on the antibiotic treatment, after discarding the upper 5 mL of supernatant, the remaining 10 mL of the middle fraction was transferred into 20 mL of fresh TB medium and incubated at 37 °C and 220 rpm for 45 min. Next, ceftriaxone (250 µg mL^−1^, Sigma‐Aldrich) was added into the culture, which was further incubated for 45–60 min. The culture (30 mL) was subsequently centrifuged at 3220 g, 4 °C for 15 min. Either the upper 25 mL of supernatant was removed, and the remaining 3 mL was collected in 1 mL aliquots for centrifugation at 16 000 g at room temperature for 20 min, or the upper 10 mL of the supernatant was removed, and the 15 mL of the middle fraction was transferred into a filtration column (Amicon Ultra Centrifugal Filter, 100 kDa MWCO, Millipore) for the centrifugation at 3220 g, 4 °C for 8 min. The minicells were then collected with ≈50–100 µL of fresh medium.

The yield of minicell isolation and the efficiency of minicell purification were quantified through brightfield microscopy imaging and plate counting assay. Minicells and bacterial cells were counted using a minimum of ten brightfield microscope images per sample to ensure statistical reliability. For the plate counting assay, serial dilutions (1:10 ratio as needed) of 100 µL of cell suspensions collected before and after each isolation step is performed. These suspensions were then plated on agar plates and incubated overnight at 37 °C. Following incubation, colony‐forming units (CFU) were enumerated to determine the concentration of viable cells as CFU mL^−1^.

The GFP expression levels of minicells were analyzed after overnight incubation in a TB medium at pH 7.4, 6.5, and 4.6. Fluorescence images were captured at 100 × magnification in the FITC channel (λex = 495 nm; λem = 519 nm), and fluorescence intensity was quantified using ImageJ software. For each pH condition, more than 60 minicells were analyzed for accurate quantification.

### Motility Analysis

Minicells were placed into a custom‐designed microchannel fabricated by assembling polymethyl methacrylate (PMMA) pieces and double‐sided adhesive films, which were cut to specific dimensions using a CO_2_ laser cutter (PLS6.150D, Universal Laser Systems) and subsequently mounted on a cover glass. For the motility characterization of minicells and minicell biohybrids, at least five videos for each sample were recorded. The video acquisition was performed using an inverted optical microscope equipped with a 100x oil immersion objective lens (Zeiss Axio Observer A1, Carl Zeiss). The tracking of minicells, including the analysis of 2D trajectories and the average swimming speed, was conducted using MATLAB R2020a (MathWorks), employing a custom‐developed tracking code.

### Fabrication of Minicell Biohybrids

The minicell biohybrids were fabricated based on the high‐affinity interaction between streptavidin and biotin. Prior to the formation of minicell biohybrids, biotin staining was performed to verify the presence of biotin on the minicell surface. Minicells suspended in fresh TB medium were incubated with fluorescently labeled streptavidin (25 µg mL^−1^, Alexa Fluor 647 Streptavidin, Thermo Fisher Scientific) under standard culture conditions for 45 min. After incubation, they were washed three times with fresh medium by centrifugation at 16 000 g for 5 min. The biotin staining was then analyzed using fluorescence imaging, acquired with an inverted fluorescence microscope (Eclipse Ti‐E, Nikon) equipped with a 100x oil immersion objective. Following the confirmation, the fabrication of minicell biohybrids was conducted by incubating minicells suspended in a TB medium with streptavidin functionalized MNPs (25 µg mL⁻¹, CD Bioparticles) at 37 °C, 220 rpm for 1 h. After incubation, minicell‐biohybrids were washed three times by centrifugation at 16 000 g for 5 min and gently resuspended for further analysis.

### Characterization of Minicell Biohybrids

The structural characteristics of the minicell biohybrids were examined using SEM. Minicell biohybrids were first deposited onto silicon wafers and allowed to settle for 30 min. They were then fixed with 2.5% (v/v) glutaraldehyde (Sigma‐Aldrich) in distilled water for 30 min at 4 °C. After fixation, dehydration was carried out using graded concentrations of ethanol (25%, 50%, 75%, 90%, and 100%), with each step lasting for 5 min. Following ethanol dehydration, chemical drying was performed with hexamethyldisilazane (HMDS, Sigma‐Aldrich) in ethanol at increasing concentrations (33%, 50%, 67%, and 100% HMDS). The samples were then left to dry overnight in a fume hood. Finally, a 10 nm gold layer was sputtered onto the samples using a Leica EM ACE600 sputter coater (Leica Microsystems) before imaging. SEM images of the minicell biohybrids were captured using a Zeiss Ultra 550 Gemini (Carl Zeiss Inc.), operated at an accelerating voltage of 3 keV with an in‐lens detector. Pseudocoloring of the SEM images was performed using Adobe Photoshop software to enhance visual contrast and detail. Magnetic characterization of minicells, MNPs, and minicell biohybrids was performed at room temperature using VSM (EZ7, MicroSense) with maximum 1.8‐T magnetic field strength. Zeta potential measurements for minicells and minicell biohybrids, which were suspended in a TB medium at neutral pH, were acquired using Zetasizer Ultra (Malvern‐Panalytical) at 25 °C.

### Magnetic Guidance of Minicell Biohybrids

The magnetic control of minicell biohybrids was conducted using a custom setup built with two circular permanent magnets positioned at a specific distance to produce a uniform magnetic field of ≈26 mT. Additionally, their magnetic steering was performed by an in‐house built electromagnetic coil setup, which can generate magnetic fields up to 20 mT in three dimensions with five coils. In this study, a uniform magnetic field of 10 mT was applied in the xy‐plane with varying directions to guide the minicell biohybrids. Both custom‐built magnetic setups were integrated with the inverted microscope to facilitate real‐time observation and control of biohybrids.

### Drug Loading in Minicells and Minicell_DOX_ Biohybrid Formation

Drug loading into minicells was carried out by incubating 3.5 × 10⁸ cells per mL with DOX (Sigma‐Aldrich) dissolved in a sterile saline solution. The minicells were exposed to varying concentrations of the drug, ranging from 10 to 200 µg mL^−1^, for incubation periods of 1 to 48 h at 30 °C and 150 rpm. Following the incubation, the minicells were washed at least three times with fresh saline by centrifugation at 40 000 g for 5 min to remove the free drug.

To prepare minicell_DOX_ biohybrids, the minicell_DOX_ were incubated with streptavidin‐functionalized MNPs (25 µg mL⁻¹) in the TB medium, following the biohybridization procedure described previously. After incubation, the minicell_DOX_ biohybrids were washed three times with fresh medium and gently resuspended for further applications.

### Drug Extraction from Minicells and Quantification

DOX was extracted from the loaded minicells by performing five cycles of vortexing and sonication using a 97 mm hydrochloric acid‐isopropyl alcohol (HCl‐IPA) solution, as described in.^[^ ^22^ ^]^ After the extraction sequence, the samples were then diluted with an equal volume of water, and the process was repeated for five cycles. Following extraction, the suspension was centrifuged at 40 000 g for 5 min to precipitate any cellular debris, and the supernatant was collected for drug quantification. The concentration of DOX loaded into minicell was quantified by measuring the fluorescence intensity of the collected supernatant using the microplate reader (λex = 485 nm; λem = 595 nm).

### Drug Release

Minicell_DOX_ (0.1 mL), previously incubated overnight with DOX at a concentration of 40 µg mL^−1^, was loaded into the mini dialysis devices (Slide A Lyzer MINI Dialysis Device, 10K MWCO, ThermoFischer Scientific), which were immersed in PBS (1.3 mL) at pH 7.4, 6.5 and 4.6. The pH‐sensitive release of DOX was conducted at 37 °C and 150 rpm. At specified time intervals, samples were collected from the dialysis device, and the withdrawn volume was replaced with fresh buffer at the corresponding pH. The amount of DOX released over time was quantified by measuring fluorescence intensity as previously described.

### Cell Culture

SK‐BR‐3 human breast cancer cells (HTB‐30, American Type Culture Collection) were cultured in modified McCoy's 5A medium (Sigma–Aldrich) supplemented with 10% fetal bovine serum (Gibco) and 1% penicillin/streptomycin (Gibco) at 37 °C and 5% CO_2_ in 75 cm^2^ polystyrene cell culture flasks within a humidified incubator. Once they reached ≈80% confluency, the cells were subcultured twice a week using 0.25% trypsin/ EDTA (Gibco) for cell detachment. For the experiments, cells were seeded with antibiotic‐free media into either 96 well plates (tissue culture‐treated; Avantor) or Ibidi µ‐slide 8 well (Ibidi GmbH) plates at a concentration of 10^5^ cells mL^−1^, depending on the experimental procedure. The cells were then incubated for at least 24 h to allow adherence and achieve confluency.

### Cellular Viability

The viability of SK‐BR‐3 cells was measured after their 24 h incubation with minicell biohybrids, minicell_DOX_ biohybrids with and without an external magnetic field (prepared by incubating minicells overnight with 40 µg mL^−1^ DOX prior to magnetic particle conjugation), and free DOX at an equivalent concentration of release at pH 6.5. For the minicell_DOX_ biohybrids subjected to magnetic control, a 10 mT uniform magnetic field with changing directions is applied for 5 min using an electromagnetic coil setup to steer minicell biohybrids after introducing them to cancer cells. Following the 24 h incubation, cell viability was assessed using a colorimetric cell counting assay with the CCK‐8 kit (Abcam), performed according to the manufacturer's instructions. For the visual detection of cellular viability, a parallel set of SK‐BR‐3 cells was stained using a Live/Dead Cell Imaging Kit (Invitrogen) according to the manufacturer's instructions. The green fluorescence (λex = 488 nm; λem = 515 nm) in live cells and red fluorescence (λex = 570 nm; λem = 602 nm) in dead cells were captured using the Nikon Eclipse Ti‐E fluorescent microscope.

### Statistical Analysis

All data were presented as the mean ± standard deviation of a minimum of three measurements. Statistical analysis was performed using one‐way analysis of variance (ANOVA), followed by Tukey's and Dunn's multiple comparisons tests, with a P‐value of less than 0.05 considered statistically significant. The statistical tests and graph plotting were performed using GraphPad Prism 7.0 (GraphPad Software Inc.). Additional data and video analyses were conducted using MATLAB 2020a, MS Excel and Fiji ImageJ.

## Conflict of Interest

The authors declare no conflict of interest.

## Author Contributions

S.F.B. and M.B.A. contributed equally to this work. S.F.B. and M.B.A. designed the study and participated in experimental procedures, data collection, data analysis, and manuscript writing. I.K. and V.S. designed and created the minicell‐producing bacterial strain and assisted with experimental procedures and manuscript writing. M.S. participated in the study design, discussions, study supervision, and manuscript writing.

## Supporting information



Supporting Information

Supplemental Movie 1

Supplemental Movie 2

Supplemental Movie 3

Supplemental Movie 4

Supplemental Movie 5

## Data Availability

The data that support the findings of this study are available from the corresponding author upon reasonable request.
