# Supplementary material for: Bacterial Minicell‐Based Biohybrid Sub‐micron Swimmers for Targeted Cargo Delivery
Source: Adv Sci (Weinh). 2025 Jun 27;12(36):e05538. doi: 10.1002/advs.202505538 (PMC12462955; doi:10.1002/advs.202505538)
Supplement: Supplementary file 1 — Supporting Information [file ADVS-12-e05538-s002.pdf]

ADVANCED SCIENCE
Open Access

Supporting Information

for *Adv. Sci.*, DOI 10.1002/advs.202505538

Bacterial Minicell-Based Biohybrid Sub-micron Swimmers for Targeted Cargo Delivery

*Saadet Fatma Baltaci, Mukrime Birgul Akolpoglu, Irina Kalita, Victor Sourjik and Metin Sitti**

Supporting Information

Bacterial Minicell-based Biohybrid Sub-micron Swimmers for Targeted Cargo Delivery

*Saadet Fatma Baltaci, Mukrime Birgul Akolpoglu, Irina Kalita, Victor Sourjik, Metin Sitti**
*Corresponding author

This PDF file includes:
Figures S1 to S14
Table S1
Captions for Movies S1 to S5

Other Supplementary Materials for this manuscript include the following:
Movies S1 to S5

Supporting Figures

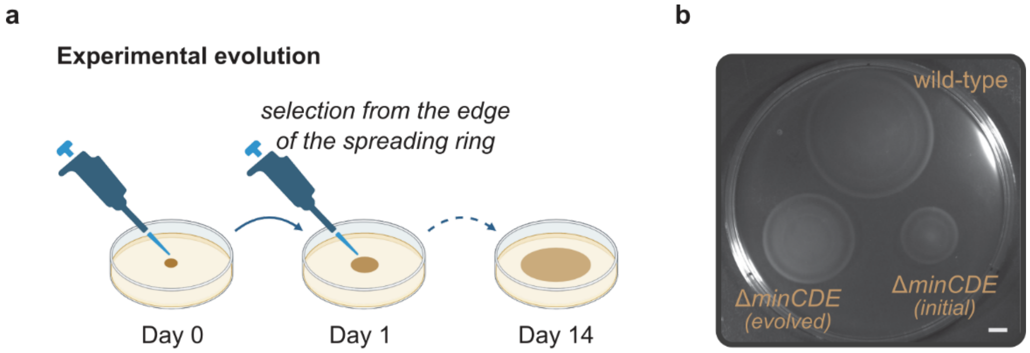

Figure S1. Experimental evolution of *Escherichia coli* Δ*minCDE* strain under selection for enhanced motility. a) A schematic of the laboratory evolution experiment in soft TB agar. Cells collected from the edge of a spreading ring were inoculated in the middle of a 0.27% soft TB agar plate and incubated at 30 °C for 15 h. This procedure was repeated over 14 days and resulted in the selection of the evolved Δ*minCDE* strain with enhanced spreading. **b)** Spreading of the wild-type (MG1655), the initial and the evolved in the course of experimental evolution Δ*minCDE* strains. The plate was kept at 37 °C for 7 h. Scale bar: 1 cm.

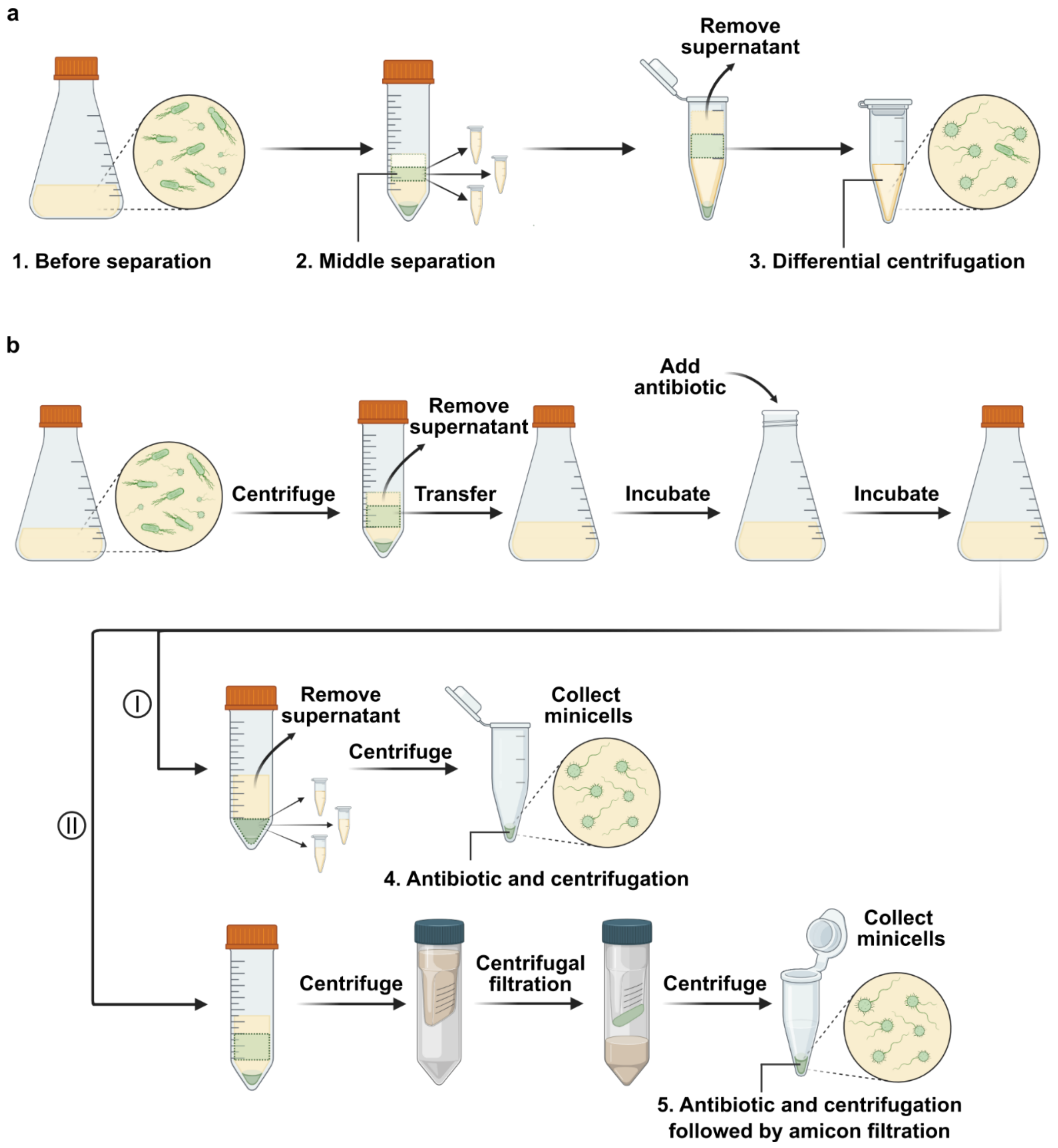

Figure S2. Detailed schematics of the minicell isolation process. a) Minicell separation with only differential centrifugation. **b)** Minicell separation and purification with an antibiotic treatment (I) followed by centrifugation and (II) followed by centrifugation and centrifugal filtration.

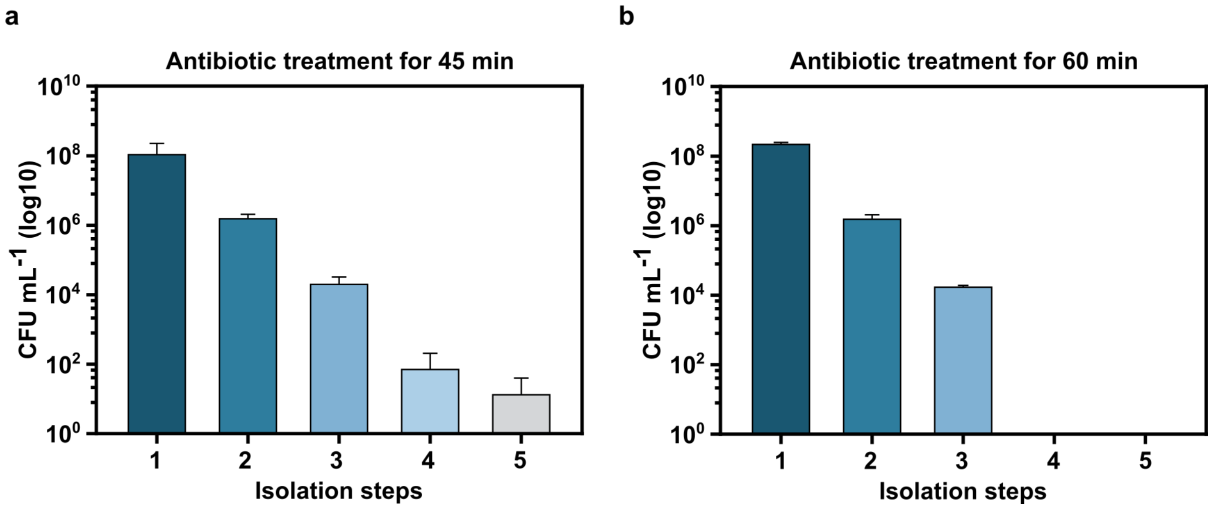

Figure S3. Minicell isolation efficiency using plate counting assay. a) Number of bacterial colonies at various stages of the isolation process: 1) before separation, 2) mid-separation, 3) differential centrifugation, 4) antibiotic treatment for 45 min followed by centrifugation, 5) antibiotic treatment for 45 min followed by centrifugation and centrifugal filtration. **b)** Number of bacterial colonies at various stages of the isolation process: 1) before separation, 2) middle separation, 3) differential centrifugation, 4) antibiotic treatment for 1 hour followed by centrifugation, 5) antibiotic treatment for 1 hour followed by centrifugation and centrifugal filtration. The error bars represent the SD of the mean for n=3.

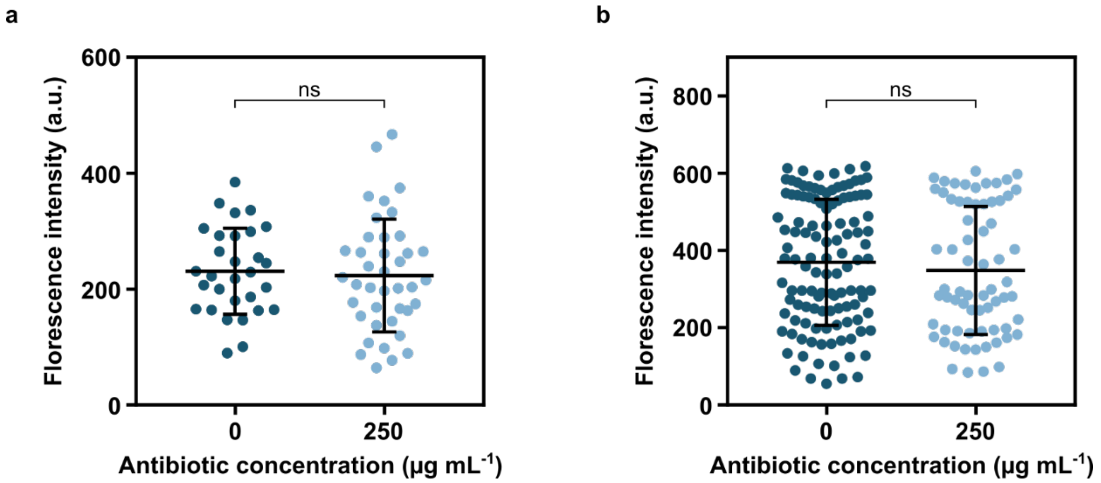

Figure S4. Effect of antibiotic treatment on protein expression in the minicell-producing strain. The figure shows the fluorescence intensity measurements for **a)** biotin staining of minicells using Cy5-labeled streptavidin, and **b)** green fluorescent protein (GFP) expression in minicells treated with and without 250 µg mL^{-1} antibiotic (ceftriaxone) during the purification process. The error bars represent the SD of the mean for n=3; ns: not significant, Student's t-test.

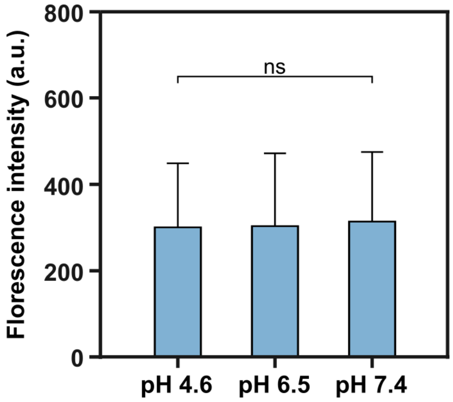

Figure S5. Functional stability of minicells under acidic conditions. The figure shows the fluorescence intensity measurements for GFP expression levels of minicells incubated overnight in TB medium at different pH values. The error bars represent the SD of the mean for the number of minicells analyzed: n = 66 at pH 4.6, n = 105 at pH 6.5, and pH = 107 at pH 7.4; ns: not significant, ANOVA followed by Tukey's multiple comparisons tests.

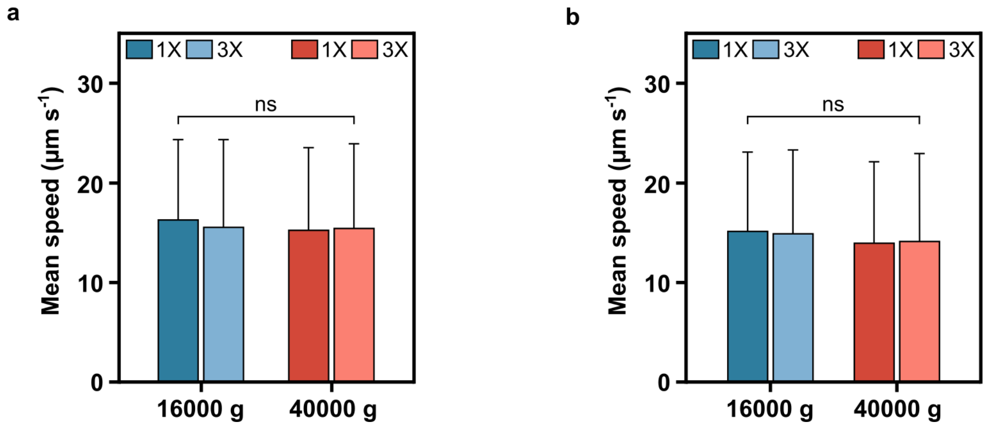

Figure S6. Effect of centrifugation force and repeated cycles on minicell motility. Mean swimming speed of minicells separated and purified with **a)** differential centrifugation and **b)** antibiotic treatment and centrifugation after applying centrifugation at 16000 g and 40000 g for one and three times. The error bars represent the SD of the mean for n=3; ns: not significant, Student's t-test.

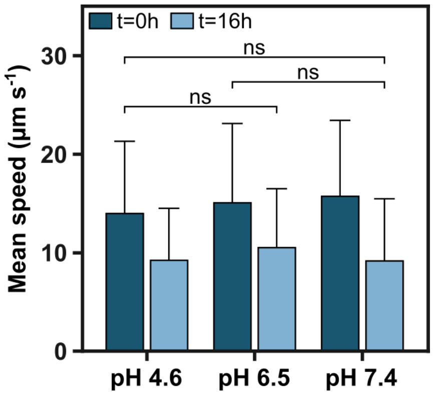

Figure S7. The effect of acidic conditions on minicell motility. Mean swimming speed of minicells incubated in TB medium at different pH values over time. The error bars represent the SD of the mean for n=5; ns: not significant, ANOVA followed by Tukey's multiple comparisons tests.

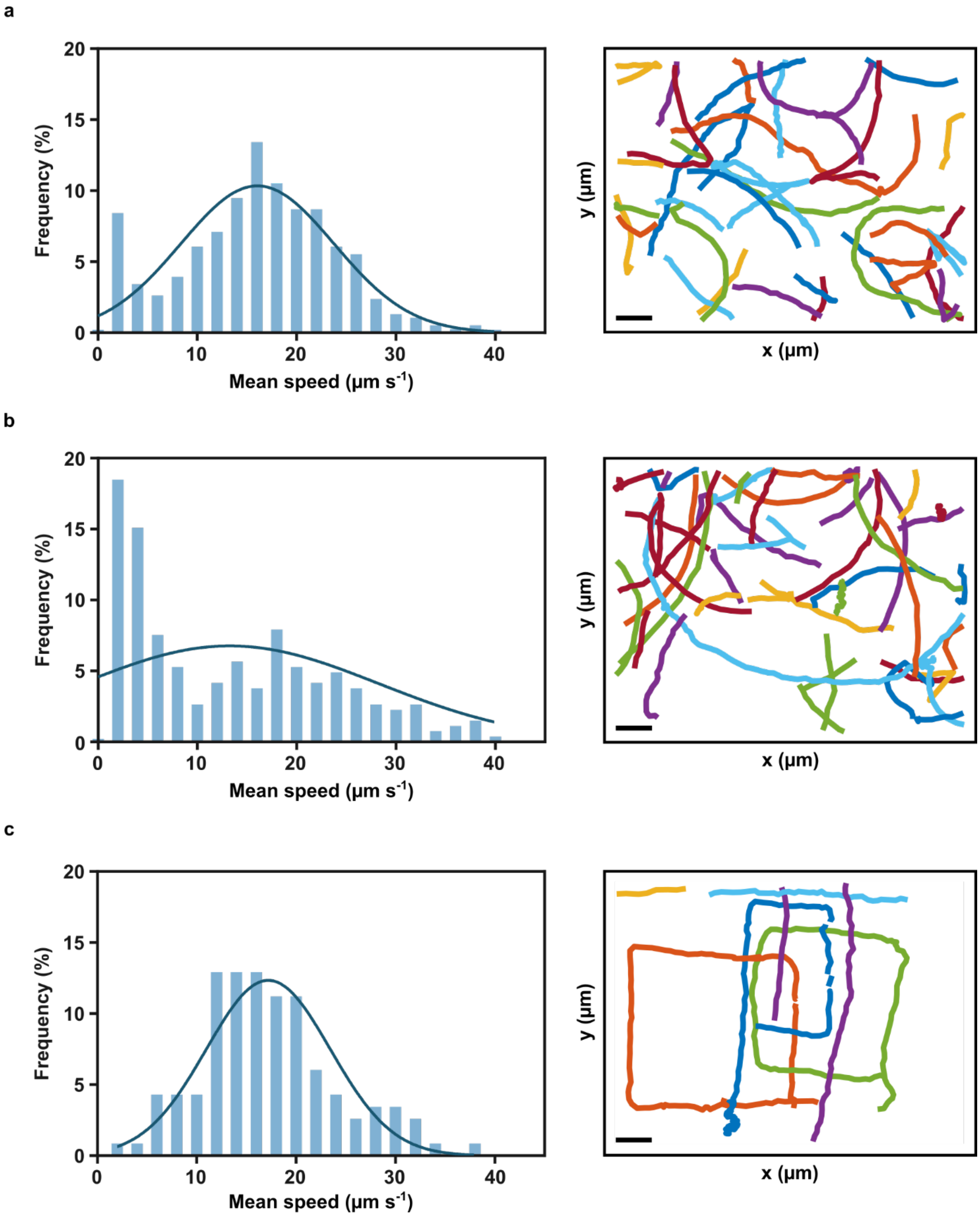

Figure S8. 2D motility analysis of minicells and minicell biohybrid swimmers (minicell biohybrids) with and without external magnetic control. Mean speed distribution and 2D swimming trajectory of **a)** bare minicells, **b)** free-swimming minicell biohybrids, **c)** minicell biohybrids under a uniform magnetic field applied by an electromagnetic coil setup, resulting in well-defined square-shaped trajectories. The data for mean swimming speed and two-dimensional trajectory analysis were obtained by tracking multiple minicells and minicell biohybrids: n = 380 for bare minicells, n = 265 for minicell biohybrids without magnetic control, and n = 116 for minicell biohybrids with magnetic control were analyzed. Scale bar: 100 μm.

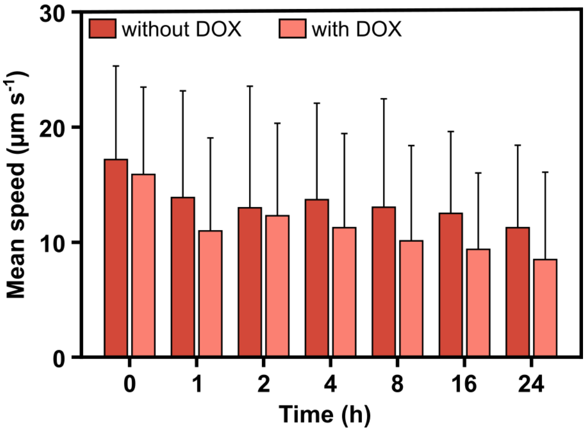

Figure S9. Motility of minicells incubated with and without doxorubicin (DOX) over time. Mean swimming speed of minicells incubated with and without 40 µg mL^{-1} DOX for 24 h. The error bars represent the SD of the mean for n=3.

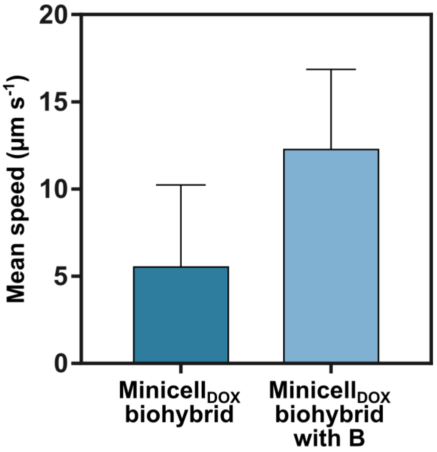

Figure S10. Motility of drug-loaded minicell (minicell$_{DOX}$) biohybrids with and without magnetic control. 2D mean swimming speeds of minicell$_{DOX}$ biohybrids with and without applied uniform magnetic field (26 mT). The error bars represent the SD of the mean for n=5.

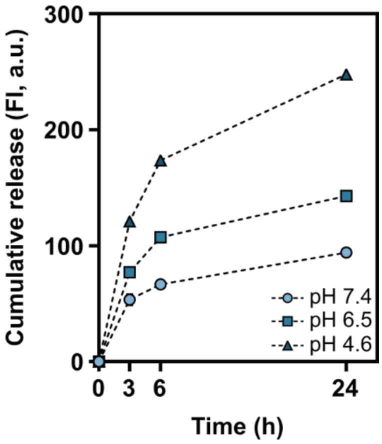

Figure S11. pH-triggered cumulative drug release from minicells within 24 hours. The figure shows the cumulative release of DOX from minicells over a 24-hour period at pH levels 7.4, 6.5, and 4.6. by highlighting the rapid release phase during the first 6 h.

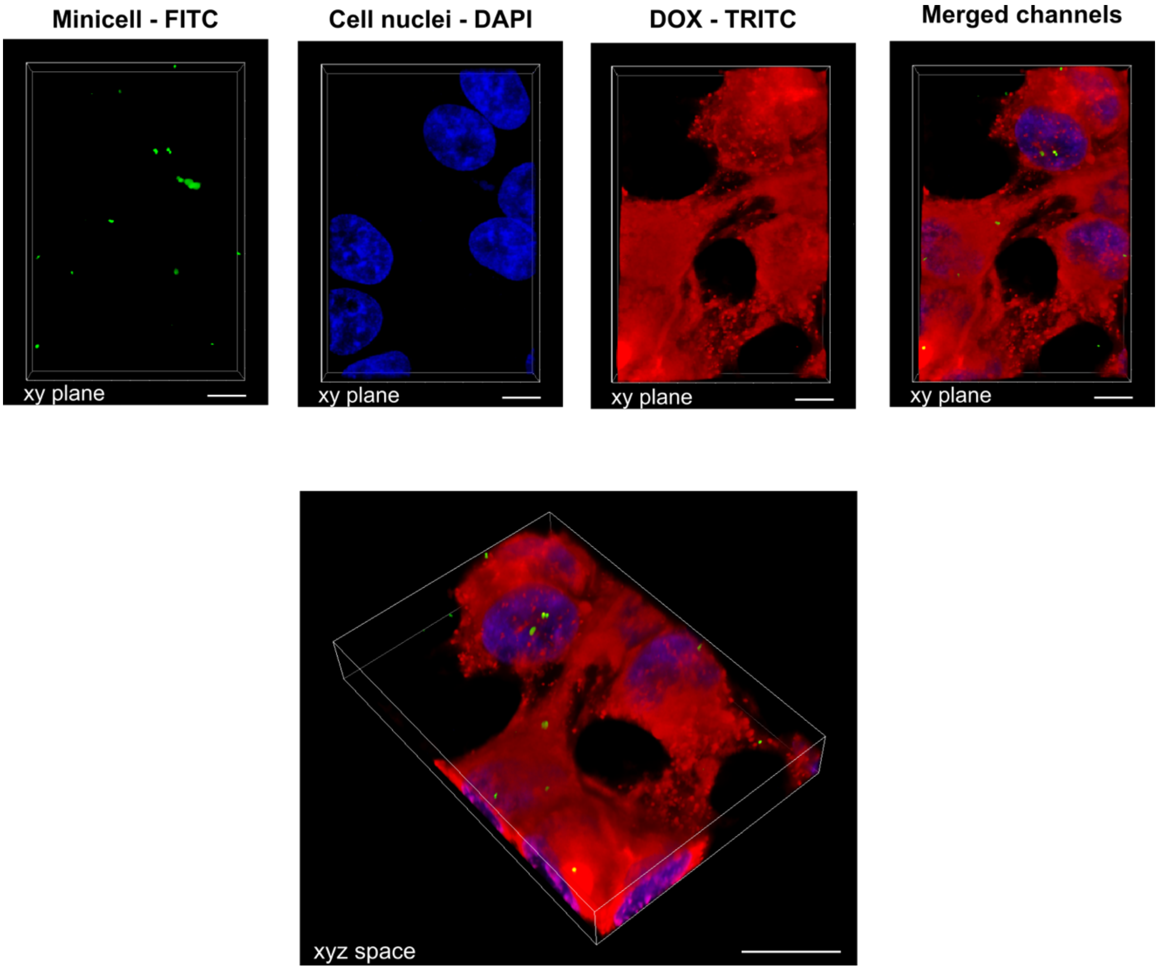

Figure S12. Confocal fluorescence microscopy images of SK-BR-3 cells incubated with DOX-loaded GFP-expressing minicell biohybrids for 24 hours. a) Two-dimensional multichannel image: cell nuclei stained with DAPI (blue), DOX fluorescence (red), and GFP-expressing minicells (green). **b)** Three-dimensional merged image showing the spatial distribution of nuclei and DOX uptake of cells, and minicell biohybrids. Scale bars: 20 µm.

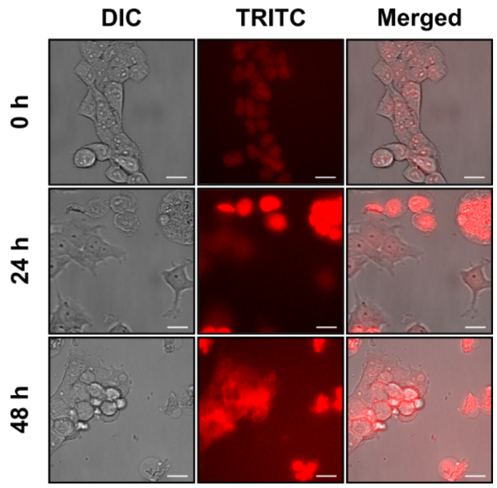

Figure S13. Fluorescence microscopy images of in vitro drug uptake of SK-BR-3 cells. The cancer cells were incubated with free DOX at an equivalent concentration of release at pH 6.5 for 0, 24 and 48 h. DOX inside the cells was shown by the red color. Scale bar: 20 µm.

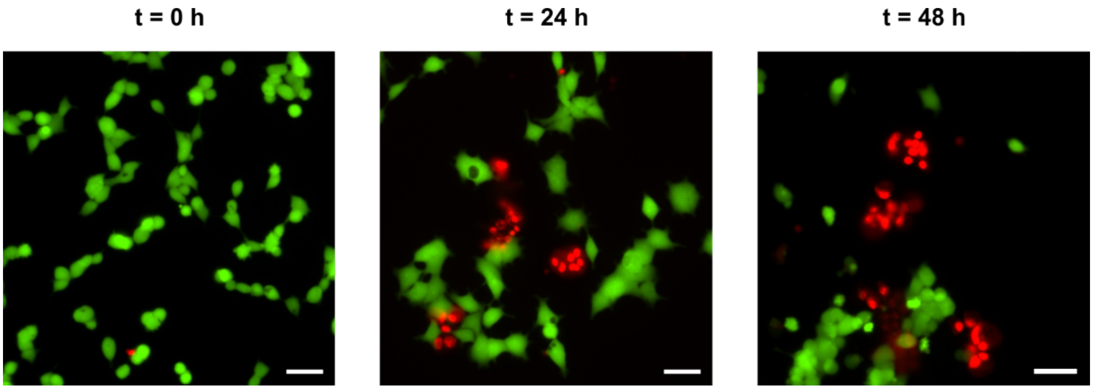

Figure S14. Live/Dead staining of SK-BR-3 cancer cells over time. The SK-BR-3 cells were treated with minicell$_{DOX}$, and live/dead staining was performed at the beginning of the experiment, as well as at 24 h and 48 h. Live cells are represented in green, while dead cells are represented in red. Scale bars: 50 μm.

Table S1. **Zeta potential measurement for minicell biohybridization.** Data are shown as mean ± SD (n = 3). An unpaired t-test with Welch's correction yielded $p = 0.1$, indicating no significant difference ($p < 0.05$).

	**Zeta potential (mV)**
**Minicell**	-17.7 ± 1.1
**Minicell biohybrid**	-20.8 ± 0.5
**MNP**	-21.4 ± 1.7

Supporting Movies

Movie S1. Swimming of minicells before and after isolation

Movie S2. Swimming of minicell biohybrids with and without magnetic control

Movie S3. Precise magnetic steering of minicell biohybrids

Movie S4. Motility of minicells after drug loading over time

Movie S5. Swimming of minicell$_{DOX}$ biohybrids with and without magnetic alignment
